# Carbogen Inhalation Therapy for Epileptic Seizures: Mechanisms, Evidence, and Future Directions

**DOI:** 10.1002/cns.70686

**Published:** 2025-12-04

**Authors:** Li Wang, Yue Liu, Wenwen Ding, Meng Yang, Qing Liu, Fuqiang Xu

**Affiliations:** ^1^ School of Medicine Jingchu University of Technology Jingmen China; ^2^ Department of Radiation Biology, Faculty of Preventive Medicine, the Ministry of Education Key Lab of Hazard Assessment and Control in Special Operational Environment The Fourth Military Medical University Xi'an China; ^3^ The Brain Cognition and Brain Disease Institute (BCBDI), Shenzhen Key Laboratory of Viral Vectors for Biomedicine, Shenzhen Institutes of Advanced Technology, Chinese Academy of Sciences, Shenzhen‐Hong Kong Institute of Brain Science‐Shenzhen Fundamental Research Institutions, NMPA Key Laboratory for Research and Evaluation of Viral Vector Technology in Cell and Gene Therapy Medicinal Products, Shenzhen Key Laboratory of Quality Control Technology for Virus‐Based Therapeutics Guangdong Provincial Medical Products Administration Shenzhen China; ^4^ University of Chinese Academy of Sciences Beijing China; ^5^ Wuhan National Laboratory for Optoelectronics Huazhong University of Science and Technology Wuhan China; ^6^ Center for Excellence in Brain Science and Intelligence Technology Chinese Academy of Sciences Shanghai China

**Keywords:** acid‐base balance, carbogen inhalation therapy, efficacy and safety, epileptic seizures

## Abstract

**Background:**

Drug‐resistant epilepsy (DRE) affects nearly one‐third of patients and remains a major unmet clinical need, despite advances in anti‐seizure medications (ASMs). Carbogen, a low‐concentration carbon dioxide (CO_2_) and oxygen (O_2_) gas mixture, has reemerged as a physiology‐based therapy owing to renewed insights into pH‐sensitive seizure mechanisms.

**Methods:**

This review summarizes preclinical and clinical evidence on carbogen inhalation for seizure control. Mechanistic data on pH‐dependent ion channels, GABAergic inhibition, and adenosine signaling were condensed, and clinical outcomes across seizure types and inhalation protocols were compared for safety and efficacy.

**Results:**

Carbogen induces short‐term respiratory acidosis, lowering brain extracellular pH to activate acid‐sensitive ion channels (e.g., ASIC1a and NaV1.2), enhance GABAergic inhibition, and boost adenosine signaling. These effects are rapid, reversible, and distinct from conventional ASMs. Preclinical studies show strong seizure suppression, especially under hyperventilation‐induced alkalosis. Clinically, brief low‐dose inhalation (5% CO_2_ for ≤ 3 min) is effective and well tolerated in focal and absence seizures, whereas earlier high‐concentration protocols caused adverse effects. Efficacy varies by seizure type: benefits in nonconvulsive status epilepticus (NCSE) are limited, and the CARDIF trial found no advantage for pediatric febrile seizures. Ongoing work, such as the CRESCENT trial, is exploring carbogen as an adjunct therapy for pediatric convulsive status epilepticus (CSE).

**Conclusions:**

Evidence supports carbogen's therapeutic promise and highlights the need for precise patient selection, controlled delivery, and comprehensive safety testing. Future research should develop biomarker‐guided, closed‐loop systems and test synergy with established ASMs to advance carbogen as a mechanism‐based therapy within precision epilepsy care.

## Graphical abstract

Carbogen, a low‐dose CO_2_/O_2_ mixture, modulates brain pH to suppress hyperexcitability through rapid and reversible pH‐dependent mechanisms distinct from conventional antiseizure medications (ASMs). Preclinical and small clinical studies show efficacy in focal and absence seizures, whereas variable responses in other types highlight the need for biomarker‐guided stratification and adaptive, closed‐loop delivery.

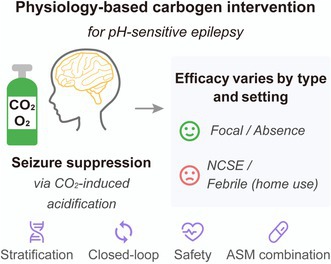

**Abbreviations**
ASIC1a: acid‐sensing ion channel 1a
ASMs: anti‐seizure medications
BZDs: benzodiazepines
CA: carbonic anhydrase
CARDIF: CARbon DIoxide against Febrile seizures
CB1: cannabinoid receptor 1
CBF: cerebral blood flow
CBZ: carbamazepine
CO_2_
: carbon dioxide
CRESCENT trial: carbogen for Status Epilepticus in Children Trial
CSE: convulsive status epilepticus
DRE: drug‐resistant epilepsy
EtCO_2_
: end‐tidal CO_2_
GFAP: glial fibrillary acidic protein
GIRK channel: G protein‐gated inwardly rectifying potassium channel
GPx4: glutathione peroxidase 4
H^+^
: protons
H_2_CO_3_
: carbonic acid
HCN channel: hyperpolarization‐activated cyclic nucleotide‐gated channel
HCO_3_^−^
: bicarbonate
HCVR: hypercapnic ventilatory response
HRV: heart rate variability
I_h_ currents: hyperpolarization‐activated cation currents
IL‐1β: interleukin‐1β
KA: kainic acid
KATP: ATP‐sensitive potassium channels
KCC2: K^+^‐Cl^−^ cotransporter 2
LEV: levetiracetam
LTG: lamotrigine
mPTP: mitochondrial permeability transition pore
NaV: voltage‐gated sodium channels
NBCe1: electrogenic Na^+^‐HCO_3_^−^ cotransporter 1
NBCn1: electroneutral Na^+^‐HCO_3_^−^ cotransporter 1
NCSE: non‐convulsive status epilepticus
NMRI mice: Naval Medical Research Institute mice
NVC: neurovascular coupling
O_2_
: oxygen
OGD: oxygen‐glucose deprivation
PaCO_2_
: arterial partial pressure of CO_2_
PBel: external lateral parabrachial nucleus
PbrO_2_
: brain tissue oxygen tension
preBötC: pre‐Bötzinger complex
PTZ: pentylenetetrazol
RCT: randomized controlled trial
ROS: reactive oxygen species
RTN: retrotrapezoid nucleus
Sst^+^
: somatostatin‐positive
SUDEP: sudden unexpected death in epilepsy
SWDs: spike‐wave discharges
TBI: traumatic brain injury
TLE: temporal lobe epilepsy
TPM: topiramate
VGSC: voltage‐gated sodium channels
VPA: valproic acid

## 1 Introduction

Epilepsy, a chronic neurological disorder marked by recurrent, unprovoked seizures, affects approximately 70 million individuals worldwide [1, 2]. Although more than 20 anti‐seizure medications (ASMs) are currently available, about one‐third of patients are refractory to treatment and develop drug‐resistant epilepsy (DRE) [3, 4]. This treatment gap underscores the urgent need for novel, rapid‐acting interventions for acute seizure control and rescue therapy, especially in emergency and out‐of‐hospital settings.

Carbogen, a fixed medical gas mixture of carbon dioxide (CO_2_) and oxygen (O_2_), has long attracted interest for its anticonvulsant potential, with clinical reports dating back to 1928 [5]. Early trials employing high CO_2_ concentrations (10%–30%) were hampered by adverse effects such as dyspnea and panic [6, 7]. More recent research has shifted attention to lower concentrations, particularly 5% CO_2_, which have demonstrated a favorable safety profile [8, 9]. Clinical trials suggest that brief inhalation of 5% CO_2_ can rapidly terminate drug‐resistant focal epilepsy and absence seizures, typically within 1–2 min, with minimal side effects [10, 11]. Unlike conventional ASMs, which rely on systemic pharmacological effects and often have a delayed onset, carbogen acts quickly by inducing transient respiratory acidosis. This direct modulation of brain pH influences neuronal activity primarily through pH‐sensitive ion channels and restoration of inhibitory tone [12, 13], enabling a rapid, reversible, and non‐pharmacologic approach to seizure control.

Despite encouraging evidence in focal and absence seizures, the clinical translation of carbogen faces several key challenges. These include limited efficacy in non‐convulsive status epilepticus (NCSE) [14, 15], uncertainties regarding optimal dosing, and unclear compatibility with existing antiseizure drug regimens. Furthermore, individual responsiveness to carbogen appears to depend on factors such as seizure phenotype, pH sensitivity, and CO_2_ chemoreception. The feasibility of consistent administration also remains constrained by practical barriers, especially in at‐home settings. For example, the CARDIF trial (NCT01370044) reported no added benefit of carbogen over placebo for pediatric febrile seizures when administered by parents, underscoring operational challenges such as reliable seizure recognition and timely delivery of treatment [16].

To bridge these gaps and optimize clinical translation, several strategic priorities have been identified. Specifically, we highlight four key directions: (1) defining and stratifying pH‐sensitive epilepsy subtypes, by leveraging biomarkers and electroclinical features to identify patients most likely to benefit from pH‐modulating therapies; (2) advancing personalized pH‐based interventions, through adaptive, closed‐loop CO_2_ delivery tailored to individual seizure dynamics and physiological responses; (3) prioritizing long‐term safety and dose optimization, through real‐time monitoring and longitudinal evaluation in diverse populations; (4) facilitating integration with existing ASMs, to assess pharmacodynamic synergy and optimize combined treatment efficacy. The ongoing multicenter CRESCENT trial (ISRCTN 52731862) is evaluating whether carbogen can serve as an effective adjunctive rescue therapy for pediatric convulsive status epilepticus (CSE) [17].

This review synthesizes current preclinical and clinical evidence on carbogen therapy in epilepsy, with emphasis on its mechanisms of action, seizure type‐specific efficacy, safety considerations, and future directions for precision‐based clinical application.

## 2 Therapeutic Effects and Mechanisms of Carbogen Inhalation

### 2.1 Acid–Base Imbalance in Seizure Pathophysiology

Acid–base homeostasis is a fundamental determinant of neuronal excitability. The brain maintains extracellular pH within a narrow range (7.35–7.45), and even minor deviations can markedly influence seizure susceptibility. Mild acidosis, whether metabolic or respiratory, generally suppresses excitability and exhibits anticonvulsant properties [12, 13]. In contrast, alkalosis, particularly when pH exceeds 7.45, has been consistently linked to increased cortical excitability and seizure propensity [18, 19].

A well‐established clinical correlate is hyperventilation‐induced alkalosis, which is routinely employed to provoke absence seizures during EEG assessments [18, 20]. Hyperventilation decreases arterial partial pressure of CO_2_ (PaCO_2_), shifting blood pH toward alkalinity. This alkalotic shift promotes hyperexcitability by modulating several pH‐sensitive ion channels, including ASIC and NaV family channels, and enhances excitatory synaptic transmission [18, 21], particularly within thalamocortical loops critical for generalized spike–wave discharges [22].

Acid–base disturbances are also frequently encountered in clinical seizure settings. For example, in neonates with hypoxic–ischemic encephalopathy, iatrogenic respiratory alkalosis from aggressive ventilation has been linked to increased seizure burden [23, 24]. Conversely, prolonged seizures or status epilepticus often induce metabolic acidosis, due to lactate accumulation and impaired oxidative metabolism [25, 26]. Interestingly, mild acidosis may be neuroprotective, potentially through enhanced GABAergic tone and inhibition of excitatory ion channel activity [27, 28].

Clinical studies further highlight the prognostic significance of pH disturbances in epilepsy. For instance, a study of status epilepticus patients found that metabolic alkalosis, but not acidosis, was associated with increased mortality and worse neurological outcomes [19]. This suggests that therapeutic correction of alkalotic states may carry clinical benefit, particularly in seizure‐prone patients.

Taken together, these findings establish respiratory alkalosis as both a mechanistic contributor to seizure generation and a rational therapeutic target. While the influence of acute pH shifts on seizure thresholds is well documented, their contribution to long‐term epileptogenesis remains unclear. Notably, emerging evidence suggests that epilepsy subtypes may differ in their reliance on pH‐sensitive pathways, a concept that will be further explored in later sections.

### 2.2 Effects of Carbogen Inhalation on Systemic Physiology

Inhalation of carbogen, a controlled mixture of CO_2_ and O_2_, has re‐emerged as a potential intervention to modulate systemic physiology and restore brain pH during seizures. Its therapeutic effects extend beyond pH correction to include respiratory stabilization and enhanced cerebral perfusion.

#### 2.2.1 Acid–Base Regulation

The principal physiological action of carbogen involves elevation of PaCO_2_, inducing a mild, reversible acidosis to correct respiratory alkalosis. CO_2_ reacts with water to form carbonic acid (H_2_CO_3_), which dissociates into protons (H^+^) and bicarbonate (HCO_3_^−^), thereby lowering extracellular pH. This acidification stabilizes hyperexcitable networks, particularly in seizure types exacerbated by alkalosis, such as absence epilepsy and febrile seizures [11, 13, 28].

Importantly, the anticonvulsant efficacy of CO_2_ is dose‐dependent. At low‐to‐moderate concentrations (5%–20%), CO_2_ induces controlled acidosis that suppresses excitability without systemic toxicity [27, 29]. In contrast, higher concentrations (25%–40%) may paradoxically increase excitability, while very high levels (> 40%) suppress activity through global central nervous system depression and respiratory inhibition [27]. These observations underscore the clinical importance of precise CO_2_ dosing to balance efficacy and safety, highlighting a key aspect in the therapeutic application of carbogen.

#### 2.2.2 Respiratory Modulation

Carbogen also contributes to seizure control by stabilizing respiratory rhythms through brainstem feedback loops. Elevated PaCO_2_ increases H^+^ concentration in the cerebrospinal fluid, activating central chemoreceptors in the medulla, particularly in the retrotrapezoid nucleus (RTN) and parafacial regions [21, 30]. This activation triggers compensatory ventilation, reversing hyperventilation‐induced hypocapnia [11].

By restoring normocapnia and buffering alkalosis, carbogen interrupts the feed‐forward loop between hypocapnia and seizure generation [31]. This is particularly relevant in children and patients with sleep‐related epilepsy, where hypocapnia‐induced instability is a recognized trigger [21, 32, 33]. Unlike sedative treatments, CO_2_ restores homeostasis without depressing consciousness or ventilation.

Importantly, carbogen's ability to stimulate rather than depress respiratory drive stands in contrast to sedative ASMs (e.g., benzodiazepines, barbiturates), which frequently precipitate respiratory depression and may require ventilatory support. This unique pharmacological and physiological profile makes carbogen an attractive adjunct for acute seizure management, especially in scenarios where maintaining spontaneous ventilation is critical [17].

#### 2.2.3 Cerebral Oxygenation and Perfusion

Carbogen also enhances cerebral perfusion and oxygenation [34], key physiological parameters often disrupted during and after seizures [35, 36]. CO_2_ is a potent cerebral vasodilator, increasing regional cerebral blood flow (CBF) and improving oxygen delivery to metabolically active brain regions [37, 38]. Compared to 100% oxygen, carbogen containing 5%–15% CO_2_ achieves superior PaO_2_, brain tissue oxygen tension (PbrO_2_), and CBF augmentation [39]. These benefits may persist beyond the inhalation window (up to 30 min), providing metabolic support during the vulnerable postictal period [40]. Collectively, these perfusion benefits may help prevent postictal hypoperfusion, often linked to postictal neuronal dysfunction and seizure recurrence, by optimizing substrate delivery and supporting recovery, aligning with neuroprotective mechanisms discussed later [41].

In summary, the systemic physiological effects of carbogen, including precise correction of alkalosis, dynamic stabilization of respiratory function, and sustained improvement in cerebral perfusion, create a supportive milieu for seizure suppression. These systemic effects form a foundation that complements the cellular and network‐level mechanisms discussed later.

### 2.3 Mechanisms of Carbogen on Acute Anticonvulsant Properties

The acute seizure‐suppressing effects of carbogen stem from CO_2_‐induced extracellular acidification, which engages multiple pH‐sensitive pathways to reduce neuronal excitability. As PaCO_2_ rises during inhalation, brain pH shifts toward mild, transient acidosis, triggering changes in ion channel activity, synaptic transmission, and neuromodulatory systems. These mechanisms operate across molecular, synaptic, and network levels to rapidly interrupt seizure activity. The following sections outline the major physiological pathways implicated in carbogen's anticonvulsant actions.

#### 2.3.1 Ion Channels and Transporters

Carbogen‐induced acidosis modifies the activity of several pH‐sensitive ion channels and transporters that govern action potential generation and synaptic excitability. One key molecular target is the acid‐sensing ion channel 1a (ASIC1a), a proton‐gated cation channel predominantly expressed in inhibitory neurons [12, 42]. Activated within a pH range of 5.8–6.8, ASIC1a facilitates Na^+^ and Ca^2+^ influx and contributes to membrane depolarization [43, 44, 45]. Rodent studies show that 10% CO_2_ inhalation rapidly terminates lethal seizures, an effect abolished in ASIC1a‐knockout (*ASIC1a* ^−/−^) mice, underscoring its mechanistic relevance [12]. Supporting this mechanism, similar effects have been observed in NaV1.1‐deficient mice (*Scn1a* ^+/−^ mice; NaV1.1 encoded by Scn1a, Dravet syndrome models), where 5% CO_2_ reduced seizure duration and increased expression of the ASIC1a protein in the hippocampus [46]. However, recent studies indicate the role of ASIC1a appears context‐dependent, varying across cell types and experimental models [12, 47, 48]. For instance, astrocyte‐specific knockdown of ASIC1a via rAAV‐mediated shRNA delivery reduces spontaneous seizures in temporal lobe epilepsy (TLE) models, whereas restoring ASIC1a expression in astrocytes increases seizure frequency, suggesting that upregulated astrocytic ASIC1a promotes seizure development [47]. This bidirectional function suggests that pH modulation can exert both pro‐ and anti‐convulsant effects depending on cellular localization and metabolic state. Recognizing these opposing mechanisms is critical for the translation of preclinical findings into rational, subtype‐specific therapeutic strategies.

In addition, voltage‐gated sodium channels (NaV) show subtype‐specific pH sensitivity [49, 50, 51]. NaV1.1, predominantly expressed in inhibitory interneurons, exhibits enhanced activity under acidic conditions, supporting inhibitory tone [52, 53]. In contrast, NaV1.2, expressed in excitatory pyramidal neurons, undergoes use‐dependent inactivation in low‐pH environments, reducing excitatory output [13, 54]. Notably, a recent study demonstrated that carbogen‐induced acidification selectively suppresses excitatory neuronal firing via NaV1.2, without affecting NaV1.1‐mediated firing in inhibitory neurons, and that these effects occur independently of ASIC1a [13]. This subtype‐specific pH sensitivity may contribute to the net inhibitory effect observed during CO_2_ exposure, offering a plausible mechanism for its seizure‐suppressive action.

Other acid‐sensitive channels include ATP‐sensitive potassium (KATP) channels, which hyperpolarize neurons under energy stress [55], and the K^+^‐Cl^−^ cotransporter 2 (KCC2), which may be upregulated by CO_2_ to restore Cl^−^ homeostasis and support GABAergic inhibition in epileptic tissue [56, 57, 58]. Together, these ion channel and transporter adaptations contribute to membrane stabilization during seizures.

#### 2.3.2 Neurotransmitter Systems and Synaptic Transmission

Carbogen‐induced acidification modulates excitatory and inhibitory neurotransmission. One key mechanism is the inhibition of glutamatergic transmission via pH‐sensitive suppression of NMDA receptor activity. Acidification reduces NMDA receptor open probability and Ca^2+^ permeability, limiting excitotoxicity during seizures and status epilepticus [59, 60, 61]. Concurrently, mild acidosis enhances GABA_A_ receptor–mediated inhibition, increasing receptor open time and current amplitude, thereby stabilizing inhibitory tone [62, 63, 64]. This dual action, suppressing excitation and enhancing inhibition, supports the rapid seizure termination observed with CO_2_ inhalation in both clinical and preclinical studies [10, 29].

Adenosine, a well‐established endogenous anticonvulsant, is also regulated by extracellular pH. Acidosis promotes adenosine accumulation via reduced reuptake and increased ATP degradation [65, 66]. Adenosine activates A1 receptors, suppressing glutamate release and promoting neuronal hyperpolarization via G protein‐gated inwardly rectifying potassium (GIRK) channels [67, 68, 69]. However, excessive adenosine may suppress respiratory drive, particularly in sudden unexpected death in epilepsy (SUDEP)‐prone conditions, requiring careful therapeutic balance [70].

CO_2_ also indirectly modulates neurotransmission through its effects on synaptic pH buffering, astrocyte‐neuron interactions, and vesicle dynamics. Acidification alters synaptic cleft pH, affecting Ca^2+^ entry, vesicle release probability, and receptor kinetics [12, 13, 69]. These secondary effects further dampen network excitability and promote seizure termination.

In summary, carbogen modulates neurotransmission through multiple coordinated pathways, including NMDA inhibition, GABA_A_ potentiation, and enhanced adenosine signaling, each contributing to rapid seizure suppression. These acute mechanisms are summarized in Table 1, which consolidates key molecular targets of CO_2_ across diverse seizure models and experimental conditions.

**TABLE 1:** Mechanistic targets of carbogen's acute anticonvulsant effects.

Mechanism type	Target/pathway	References	Research subject	Protocol	Main effects
Ion channels	ASIC1a (neuronal)	Ziemann 2008 [12]	KA/PTZ‐induced seizures (mouse)	PcTx1 inhibition/10% CO_2_ inhalation	ASIC1a activation by CO_2_ reduced seizures; inhibition worsened severity
	ASIC1a	Lu 2025 [46]	Dravet (*Scn1a* ^+/−^ mouse)	5% CO_2_ inhalation	CO_2_ increased ASIC1a in hippocampal neurons; reduced seizures and damage
	ASIC1a (astrocytic)	Yang 2016 [47]	Pilocarpine model (mouse)	rAAV‐shRNA knockdown or overexpression in astrocytes	Inhibiting astrocytic ASIC1a reduced spontaneous seizures; overexpression increased seizure frequency
	NaV1.2 (excitatory)	Hatch 2023 [13]	Thermogenic seizure model (*Scn1a* ^+/−^ mouse)	5% CO_2_ exposure	CO_2_ suppressed excitatory neuron firing by inhibiting NaV1.2; effect occurred independently of ASIC1a
	KATP channel	Niaki 2008 [55]	NMRI mice	Mouth breathing/CO_2_ retention	CO_2_ retention increased seizure threshold; effect blocked by glibenclamide
Ion transporters	KCC2	Zions 2020 [56]	KCC2‐mutant rat	Heat stress/CO_2_ exposure	High CO_2_ levels helped restore GABAergic inhibition and prevent seizures
	KCC2	Uwera 2015 [58]	Rat hippocampal slice	Furosemide and CO_2_ co‐modulation	CO_2_ restored Cl^−^ extrusion and intracellular pH homeostasis
Neurotransmission	GABA/Glutamate	Shi 2017 [29]	KA‐induced seizure (rat)	5% CO_2_ inhalation; cortical pH, neurotransmitter levels, and ECoG measured	Increased GABA, reduced glutamate, suppressed discharges
	GABA_A_ receptor	Brosnan 2008 [59]	Rat hippocampal slice	CO_2_ perfusion	CO_2_ potentiated GABAA‐mediated inhibition, suppressed seizures
	NMDA receptor	Pasternack 1996 [62]	Xenopus NR1/NR2A system	pH‐controlled perfusion	Acidosis inhibited NMDA receptor currents dose‐dependently
Neuromodulators	Adenosine (A1R)	Dulla 2005 [69]	Rat hippocampal slice	5% CO_2_ in aCSF	CO_2_ increased adenosine, reduced synaptic excitation
	Adenosine (A1R)	Dulla 2009 [67]	A1R‐knockout mouse hippocampal slice	5% CO_2_ in aCSF	CO_2_ effect was abolished in A1R‐ knockout mice
	Adenosine‐SUDEP interaction	Purnell 2024 [70]	*Adk* ^*+/−*^ mouse model	Genetic/pharmacological manipulation of adenosine metabolism	Excess adenosine linked to respiratory suppression
	Serotonin (arousal)	Faingold 2023 [71]	SUDEP rodent models	Review	Serotonin promotes arousal; counters adenosine‐induced depression
Respiratory circuits	Phox2b RTN neurons	Guyenet 2015 [72]	Brainstem chemosensory	Hypercapnia	CO_2_ activated RTN neurons; restored breathing
	Sst^+^ parafacial neurons	Cleary 2021 [73]	Sst‐Cre mice	10% CO_2_, voltage‐clamp	Identified as pH/CO_2_ sensors regulating rhythmic breathing
	5‐HT raphe neurons → PBel	Faingold 2023 [71]	SUDEP models, human case data	Review	CO_2_ activates serotonergic arousal circuit
Forebrain circuits	Hippocampus	Schuchmann 2006 [28]	Febrile seizure (rat)	Treated with 5% CO_2_	CO_2_ suppressed hippocampal hyperexcitability via I_h_ inhibition
	Basolateral amygdala	Ziemann 2009 [74]	WT/ASIC1a‐knockout mice	CO_2_ inhalation, pH buffering, ASIC1a manipulation	CO_2_‐induced acidification activated ASIC1a in the amygdala, enhancing GABAergic tone and suppressing excitability
	Neocortex	Tolner 2011 [10]	Cortical models	CO_2_ inhalation	Reduced discharges in frontotemporal and sensorimotor cortex

*Note:* Pathway integration is summarized in the main text and Figure 1.

Abbreviations: A1R‐knockout mice, adenosine A1 receptor knockout mice; KA, kainic acid; NMRI mice, Naval Medical Research Institute mice; PBel, external lateral parabrachial nucleus; PTZ, pentylenetetrazol; RTN, retrotrapezoid nucleus; Sst^+^, somatostatin‐positive.

#### 2.3.3 Respiratory and Arousal Circuits

Beyond local effects, carbogen may influence seizure control through brainstem circuits involved in respiratory rhythm and arousal. These circuits include the RTN, pre‐Bötzinger complex (preBötC), and medullary raphe nuclei, which contain pH‐sensitive neurons essential for ventilatory homeostasis [72, 75, 76].

Phox2b‐expressing neurons in the RTN serve as central chemoreceptors and are robustly activated by elevated CO_2_, driving respiratory compensation through increased ventilation [72, 77]. Somatostatin‐positive (Sst^+^) parafacial neurons, also located in the ventral medulla, provide tonic excitatory drive and are highly sensitive to CO_2_, further supporting rhythmic breathing at baseline [73]. These circuits form a neurophysiological substrate through which carbogen may prevent postictal respiratory suppression, a critical contributor to SUDEP [77, 78].

Medullary serotonergic neurons, especially those in the dorsal and median raphe nuclei, also play a key role in respiratory regulation and arousal recovery [79]. These neurons project to the external lateral parabrachial nucleus (PBel) and forebrain, where they promote arousal in response to hypercapnia via 5‐HT_2_A receptor signaling [71, 80]. Activation of this serotonergic‐parabrachial pathway may aid postictal autoresuscitation, particularly in sleep‐related forms of epilepsy characterized by impaired arousal.

However, seizures can impair chemosensory function. In Dravet syndrome models, brainstem CO_2_ sensitivity is reduced, limiting compensatory ventilation and potentially diminishing carbogen efficacy [21, 32]. Human studies show interindividual variability in ventilatory responses to CO_2_ [81, 82], suggesting that personalized monitoring and risk stratification may be needed when using carbogen in high‐risk populations. To address these limitations, pharmacological strategies aimed at enhancing the ventilatory response to CO_2_, such as serotonergic augmentation, are under investigation and may complement carbogen therapy in selected patients [83, 84].

#### 2.3.4 Forebrain and Network‐Level Mechanisms

Carbogen also modulates forebrain excitability and network synchronization. In the hippocampus, 5% CO_2_ reduces hyperexcitability via inhibition of hyperpolarization‐activated cyclic nucleotide‐gated (HCN) channel‐mediated hyperpolarization‐activated cation (I_h_) currents and normalization of glutamatergic tone [28]. In the basolateral amygdala, acidification enhances GABAergic inhibition through ASIC1a activation, contributing to seizure suppression [74]. Notably, the amygdala also plays a dual role in autonomic control and has been implicated in seizure‐induced apnea and SUDEP, underscoring its relevance for both ictal and postictal physiology [85, 86].

Paralimbic areas, such as the piriform and entorhinal cortices, are highly pH‐sensitive and act as seizure amplifiers [87, 88]. Their CO_2_ responsiveness suggests potential involvement in propagation dynamics, though direct evidence is limited [89].

Extending to neocortical and thalamocortical systems, EEG studies demonstrate that 5% CO_2_ inhalation suppresses epileptiform discharges in frontotemporal and sensorimotor cortices, likely through reduced cortical synchronization [10]. Recent work in WAG/Rij rats demonstrates that hyperventilation‐induced alkalosis triggers absence seizures by activating pH‐sensitive neurons in the intralaminar thalamic nuclei, indicating a key role for thalamocortical pH sensitivity in seizure initiation [18].

Astrocytes complement neuronal responses to CO_2_ by buffering extracellular K^+^, regulating pH, and modulating gliotransmission [26]. Acidification activates astrocytic ASIC1a, dampening hyperexcitability [47]. In Dravet models, CO_2_ exposure reduces astrogliosis and neuronal injury [46], suggesting a supportive glial role in seizure control.

**FIGURE 1:**
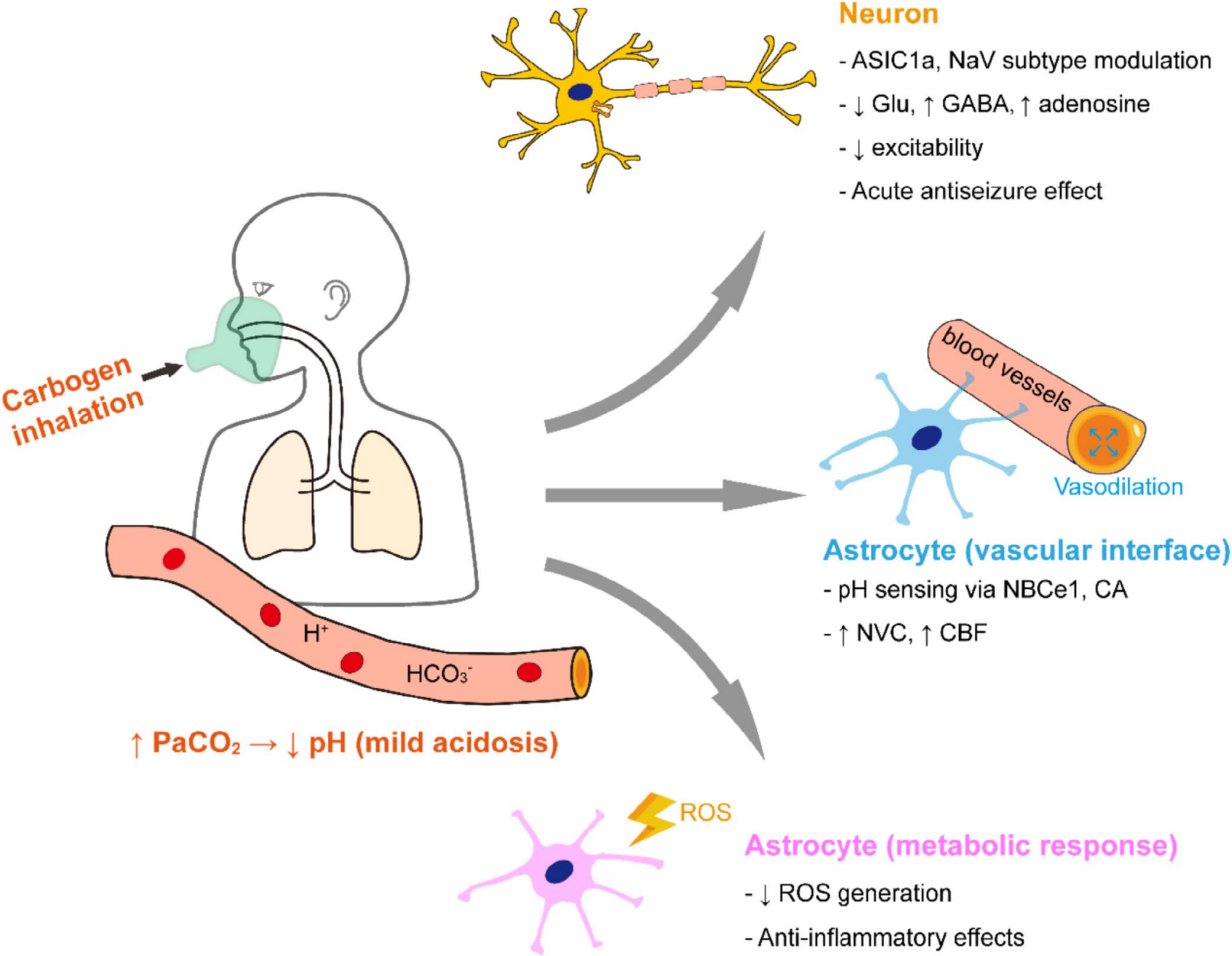
Integrated mechanisms of carbogen's anticonvulsant and neuroprotective actions. Carbogen inhalation increases arterial CO_2_ (PaCO_2_), leading to mild, transient brain acidosis. This engages pH‐sensitive neuronal and glial pathways that suppress seizures and enhance tissue resilience. In neurons, acidification activates ASIC1a and modulates NaV subtypes, reduces glutamate release, enhances GABAergic and adenosinergic signaling, and lowers excitability. At the vascular interface, astrocytes sense pH shifts via NBCe1 and carbonic anhydrase (CA), promoting neurovascular coupling (NVC) and increasing cerebral blood flow (CBF). In metabolically reactive astrocytes, acidosis reduces ROS production and inflammation in a dose‐dependent manner, contributing to long‐term neuroprotection. These converging mechanisms underpin carbogen's dual action as a fast‐acting and potentially disease‐modifying therapy in epilepsy.

Collectively, these findings reveal that carbogen exerts rapid antiseizure effects through an orchestrated network of pH‐sensitive mechanisms. These include ion channel modulation, synaptic transmission reshaping, respiratory stabilization, and glial‐neuronal interactions. Figure 1 provides a conceptual synthesis of these pathways across molecular, cellular, and systems levels. Table 1 summarizes experimental evidence supporting these mechanisms across multiple preclinical epilepsy models.

### 2.4 Mechanisms of Carbogen on Neuroprotection

In addition to its acute seizure‐suppressive effects, carbogen may confer long‐term neuroprotection by mitigating excitability‐related plasticity, dampening neuroinflammatory cascades, and restoring metabolic homeostasis. These mechanisms target key drivers of epileptogenesis and postictal vulnerability.

#### 2.4.1 Modulation of Seizure‐Induced Network Plasticity

Seizures induce persistent alterations in synaptic transmission, ion channel expression, and circuit excitability that promote chronic seizure susceptibility and disease progression [90, 91]. Although direct evidence linking carbogen to suppression of long‐term plasticity is limited, findings from febrile seizure models suggest CO_2_ exposure may attenuate maladaptive remodeling. Specifically, 5% CO_2_ inhalation prevented the postictal upregulation of HCN channels and cannabinoid receptor 1 (CB1) in the hippocampus [28], two molecules associated with heightened excitability and impaired network stability [92, 93, 94].

In parallel, mechanistic studies implicate bicarbonate transport in pH‐sensitive plasticity. Deletion of the electroneutral Na^+^‐HCO_3_^−^ cotransporter 1 (NBCn1), which is responsible for promoting intracellular alkalosis, increased seizure resistance and reduced excitability, suggesting that intracellular alkalosis facilitates epileptogenic adaptation [95].

Together, these findings suggest that CO_2_‐induced acidosis may counteract seizure‐triggered plasticity by disrupting permissive pH conditions for maladaptive remodeling. However, this hypothesis requires validation in chronic epilepsy models, ideally incorporating longitudinal assessments of gene expression, circuit function, and behavior.

#### 2.4.2 Suppression of Neuroinflammation and Oxidative Stress

Neuroinflammation and astrocyte reactivity are major drivers of seizure‐induced neuronal injury [96]. Preclinical studies show that carbogen suppresses proinflammatory cascades (e.g., NF‐κB, MAPK), reduces microglial activation, and stabilizes the blood–brain barrier [97, 98]. Astrocytes may also contribute to carbogen's modulatory effects. In TLE models, knockdown of astrocytic ASIC1a reduced spontaneous seizures, implicating pH‐sensitive glial pathways in excitability control [47]. In Dravet syndrome models, 5% CO_2_ exposure reduced hippocampal injury and attenuated reactive astrocytosis [46], suggesting potential structural neuroprotection beyond acute seizure termination.

Beyond the anti‐inflammatory effects, carbogen‐induced acidosis also reduces reactive oxygen species (ROS) generation and protects mitochondrial function by attenuating lipid peroxidation and improving redox homeostasis [25, 99, 100]. Recent studies have implicated ferroptosis, a form of regulated cell death driven by lipid peroxidation and mitochondrial dysfunction, as a downstream effector of oxidative injury in epilepsy [101, 102, 103]. In seizure models, inhibition of ASIC1a attenuated ferroptosis markers and rescued neuronal viability, implicating convergent pathways that may underlie carbogen's neuroprotective effects [104].

Importantly, these effects are dose‐dependent. Hypercapnic exposure exceeding 7% CO_2_, especially under hypoxic conditions, may paradoxically promote inflammation and ROS generation [105]. These findings underscore the need for precision dosing to balance efficacy and avoid iatrogenic harm.

#### 2.4.3 Restoration of Cerebral Perfusion and Metabolic Support

Seizures impose high metabolic demands and frequently impair cerebral perfusion, resulting in transient hypoxia, glucose depletion, and mitochondrial stress, especially during prolonged or clustered seizures [25, 106, 107]. Carbogen may alleviate these deficits by restoring neurovascular coupling (NVC), improving CBF, and preserving energy homeostasis. CO_2_ functions as a potent vasodilator, acting via pH‐sensitive astrocytic mechanisms, particularly electrogenic Na^+^‐HCO_3_^−^ cotransporter 1 (NBCe1), carbonic anhydrase, and calcium exchange pathways [37]. Astrocytic CO_2_/pH sensing triggers Ca^2+^ transients and ATP release, which in turn amplify local perfusion and support gliovascular coordination during metabolic stress [47, 108]. These glial‐mediated perfusion responses are particularly relevant in postictal states, where prolonged hypoperfusion can worsen secondary injury.

Although direct data from epilepsy models are limited, findings from ischemic paradigms show that mild acidosis preserves mitochondrial integrity by reducing depolarization, stabilizing the respiratory chain, and limiting mitochondrial permeability transition pore (mPTP) opening [109, 110]. These effects suggest that carbogen may indirectly protect mitochondrial function by maintaining perfusion and reducing metabolic stress during seizures. Validation in seizure‐specific models is essential.

Carbogen exerts neuroprotective effects through multiple converging pathways: modulation of maladaptive plasticity, suppression of oxidative and inflammatory injury, and restoration of metabolic resilience via astrocyte‐mediated perfusion support (Table 2). These mechanisms extend beyond seizure suppression, positioning carbogen as a dual‐action therapy for selecting epilepsy subtypes. These multifaceted mechanisms are schematically summarized in Figure 1. Future research should focus on dose precision, biomarker‐guided stratification, and longitudinal evaluation of neuronal and glial outcomes in chronic models.

**TABLE 2:** Mechanisms underlying carbogen's neuroprotection across neurological models.

Mechanism type	Molecular/cellular target	References	Model	Protocol	Neuroprotective effect
Network plasticity	HCN channel (I_h_ currents), CB1 receptor	Schuchmann 2006 [28]	Rat febrile seizure model	5% CO_2_ inhalation during hyperthermia	Suppressed I_h_ upregulation and CB1 overexpression in hippocampus
	Na^+^‐HCO_3_^−^ cotransporter (NBCn1)	Sinning 2015 [95]	Slc4a10 knockout mouse	Field potential and pH modulation ex vivo	NBCn1 deletion reduced excitability, suggesting pH buffering limits maladaptive plasticity
Neuroinflammation and Oxidative stress	Astrocytic ASIC1a	Yang 2016 [47]	TLE mouse model (Pilocarpine)	rAAV knockdown of ASIC1a in astrocytes	Reduced spontaneous seizures after knockdown
	Astrocytic ASIC1a	Lu 2025 [46]	Dravet (*Scn1a* ^+/−^) mouse model	5% CO_2_ inhalation post‐seizure	Increased ASIC1a expression and reduced astrocyte proliferation in the hippocampus
	Ferroptosis markers (GPx4, Fe^2+^, mPTP)	Shi 2024 [104]	0‐Mg^2+^ seizure model (in vitro)	ASIC1a inhibitor exposure	Reversed ferroptosis indicators, reduced neuronal death
Perfusion and metabolism	NVC and CBF via astrocytes, NBCe1	Hosford 2022 [37]	Rat model	fMRI, voltammetry under CO_2_ manipulation	CO_2_‐induced pH shift enhanced NVC; disrupted by NBCe1 knockdown
	Mitochondrial respiratory chain function	Zhu 2019 [109]	OGD in vitro model	Acidic buffer post‐OGD	Preserved ATP levels, limited membrane depolarization and ROS
	mPTP, apoptosis	Fan 2014 [110]	Stroke rat model	20% CO_2_ inhalation post‐reperfusion	Inhibited mPTP opening, reduced neuronal apoptosis

Abbreviations: ASIC1a, acid‐sensing ion channel 1a; CB1, cannabinoid receptor 1; CBF, cerebral blood flow; GPx4, glutathione peroxidase 4; HCN, hyperpolarization‐activated cyclic nucleotide‐gated channel; I_h_ currents, hyperpolarization‐activated cation currents; mPTP, mitochondrial permeability transition pore; NBCe1, electrogenic sodium‐bicarbonate cotransporter 1; NBCn1, electroneutral sodium‐bicarbonate cotransporter 1; NVC, neurovascular coupling; OGD, oxygen–glucose deprivation; ROS, reactive oxygen species; TLE, temporal lobe epilepsy.

## 3 Current Research Landscape

### 3.1 Preclinical Studies

Preclinical studies have extensively investigated the effects of carbogen inhalation across diverse epilepsy models, uncovering key mechanistic insights and laying a foundation for clinical translation.

In a rat model of myoclonic seizures, 5% CO_2_ inhalation reduced cortical after‐discharges by approximately 75% and attenuated behavioral seizure severity. Comparable effects were observed at 10% CO_2_; however, no additional benefit was noted with higher concentrations, highlighting the sufficiency of low‐dose protocols [10]. These effects were replicated in nonhuman primates, strengthening the translational relevance [10]. Interestingly, the response to single‐pulse or bicuculline‐induced spikes was less pronounced [10], suggesting carbogen preferentially suppresses sustained high‐frequency epileptiform activity rather than transient or milder abnormal discharges.

In the kainic acid (KA) model of focal epilepsy, 5% CO_2_ inhalation delayed seizure onset, reduced frequency, and suppressed epileptiform activity in both the hippocampus and the neocortex [29], indicating potential applicability in TLE.

Carbogen also shows high efficacy in pH‐sensitive epilepsy models. In WAG/Rij rats, 5% CO_2_ inhalation reversed hyperventilation‐induced respiratory alkalosis and suppressed spike‐wave discharges (SWDs) via thalamocortical modulation [18]. In febrile seizure models, carbogen corrected alkalosis and terminated hyperthermia‐induced seizures within seconds, while also preventing long‐term hippocampal plasticity changes [28]. These results support its therapeutic relevance in pediatric seizure settings.

In models of Dravet syndrome, findings are model‐dependent. In Scn1a A1783V mutation mice, 10% CO_2_ alleviated both seizure activity and respiratory dysfunction, while 5% had minimal effect [53]. Conversely, in *Scn1a* ^+/−^ mice, even 5% CO_2_ was sufficient to reduce seizure duration, elevate hippocampal ASIC1a expression, and protect against neuronal injury [46], suggesting genotype‐specific dose responsiveness.

In acquired epilepsy models, CO_2_ retention has shown seizure‐suppressive effects. For example, in a pig traumatic brain injury (TBI) model, brief apnea‐induced hypercapnia reduced seizure duration in the injured hemisphere, regardless of whether it was applied before or after seizure induction [31]. However, the specific contributions of hypercapnia versus hypoxia remain unresolved [111].

Collectively, animal data demonstrate that low‐dose carbogen (5%) is consistently anticonvulsant in pH‐sensitive seizure models and may offer both acute and preventive benefits (see Table 3). However, optimizing dose, timing, and genotype‐specific protocols remains an area for further investigation.

**TABLE 3:** Preclinical studies on carbogen‐based respiratory interventions for seizure control.

References	Model/seizure types	Research subject	Intervention protocol	Main findings
Tolner 2011 [10]	Myoclonic (cortical stimulation)	Rat	5% CO_2_ for 5 min, pre‐stimulation	Suppressed cortical afterdischarges by ~75%
Tolner 2011 [10]	Myoclonic (cortical stimulation)	Macaque	5% CO_2_‐induced hypercapnia via hypoventilation	Suppressed cortical afterdischarges by ~70%
Tolner 2011 [10]	Focal (bicuculline‐induced)	Macaque	5% CO_2_‐induced hypercapnia	Reduced epileptiform spikes by ~25%
Shi 2017 [29]	Focal (KA‐induced)	Rat	5% CO_2_ for 1 h post‐KA	Delayed seizure onset; reduced frequency
Salvati 2022 [18]	Absence (hyperventilation‐induced)	WAG/Rij rat	5% CO_2_ + 10% O_2_ for 30 min	Reversed alkalosis, suppressed SWDs
Schuchmann 2006 [28]	Febrile (hyperthermia‐induced)	Rat	5% CO_2_ during hyperthermia	Terminated seizures in ~20 s; prevented hippocampal injury
Ohmori 2013 [53]	Genetic (Dravet)	Scn1a‐mutant mouse	5% or 10% CO_2_ for 3 min post‐induction	Only 10% CO_2_ shortened seizures significantly
Lu 2025 [46]	Genetic (Dravet)	Scn1a‐mutant mouse	5% CO_2_ for 60 min	Reduced seizure duration and neuronal injury; increased ASIC1a expression
Rodriguez Lara 2024 [31]	Post‐traumatic (TBI‐induced)	Pig	Apnea (1 min) + hypoventilation (10 min) pre‐ or post‐seizure	Reduced seizure burden ipsilaterally

Abbreviations: KA, kainic acid; SWDs, spike‐wave discharges; TBI, traumatic brain injury.

In summary, preclinical evidence supports carbogen's anticonvulsant efficacy across multiple seizure models, particularly those linked to pH dysregulation. Low‐dose (5%) CO_2_ reliably suppresses absence, febrile, and focal seizures, while higher concentrations (e.g., 10%) may be required in certain genetic contexts, such as Dravet syndrome with Scn1a mutations (see Table 3). However, effects are not universal: some seizure types respond weakly, and mechanisms remain incompletely defined. Model‐specific variability, inconsistent dose–response relationships, and limited long‐term studies highlight the need for standardized protocols and stratified designs. Future preclinical work should prioritize mechanistic dissection, dose optimization, and validation in models reflecting clinical heterogeneity.

### 3.2 Clinical Trials

Historically, the clinical application of carbogen dates back to the early 20th century. In 1928, Lennox reported that 10% CO_2_ inhalation could suppress EEG spike‐wave patterns in patients with absence seizures [5]. Subsequent studies in psychiatric cohorts showed that higher concentrations (15%–30% CO_2_) prevented electrically induced convulsions, but these levels raised concerns about safety and patient comfort [6, 7]. As a result, later research focused on evaluating lower, clinically tolerable CO_2_ concentrations, paving the way for modern trials [20].

Over the past decade, renewed interest in 5% CO_2_‐enriched carbogen has been driven by preclinical findings from the Kaila group showing that 5% CO_2_ could reverse respiratory alkalosis‐induced seizures [28, 112]. In a subsequent translational study, Tolner et al. demonstrated that 5% CO_2_ rapidly terminated electrographic seizures, typically within 1–2 min even after generalization, in patients with drug‐resistant focal epilepsy, consistent with parallel efficacy in rodent and primate models [10]. Complementing this, a pediatric study by Zou et al. demonstrated that 5% CO_2_ inhalation effectively managed hyperventilation‐induced absence seizures, suppressing SWDs without inducing discomfort [11]. These data highlight the therapeutic potential of low‐concentration carbogen, particularly for focal epilepsy and seizure types linked to respiratory alkalosis, and emphasize the need for validation in larger patient cohorts.

However, carbogen's efficacy appears to vary depending on seizure type and clinical setting. While results for focal and absence epilepsy are promising, trials in more severe or heterogeneous conditions, such as status epilepticus, have been less consistent. Forsyth et al. conducted an open‐label study of 5% CO_2_ in pediatric NCSE, finding only limited and transient effects on EEG activity, without clear clinical improvement [14]. Further analysis revealed significant inter‐individual response variability [15], underscoring both the need for personalized approaches and the complexity of CO_2_‐mediated neuromodulation in diverse seizure circuits.

Extending this research, Forsyth and colleagues launched a multicenter, double‐blind, randomized controlled superiority trial in 2024 to evaluate carbogen's efficacy as an adjunctive treatment of pediatric CSE (Carbogen for Status Epilepticus in Children Trial, CRESCENT; ISRCTN 52731862) [17]. This trial represents a critical step toward establishing evidence‐based guidelines for carbogen use in high‐severity clinical scenarios, although results are pending.

In parallel, a German team coordinated by Schuelke conducted the CARDIF trial (CARbon DIoxide against Febrile seizures; NCT01370044), a monocentric, prospective, double‐blind, placebo‐controlled, crossover study of 5% CO_2_ in febrile seizures administered at home by parents [113]. Despite a robust mechanistic rationale and the development of a parent‐operated delivery device, the trial demonstrates no significant difference in seizure termination between carbogen and placebo (oxygen) groups [16]. These findings indicate that real‐world variables, including user proficiency, delayed seizure recognition, and absence of supervised monitoring, may critically influence treatment efficacy, underscoring that biological mechanisms alone are insufficient to predict clinical success.

**FIGURE 2:**
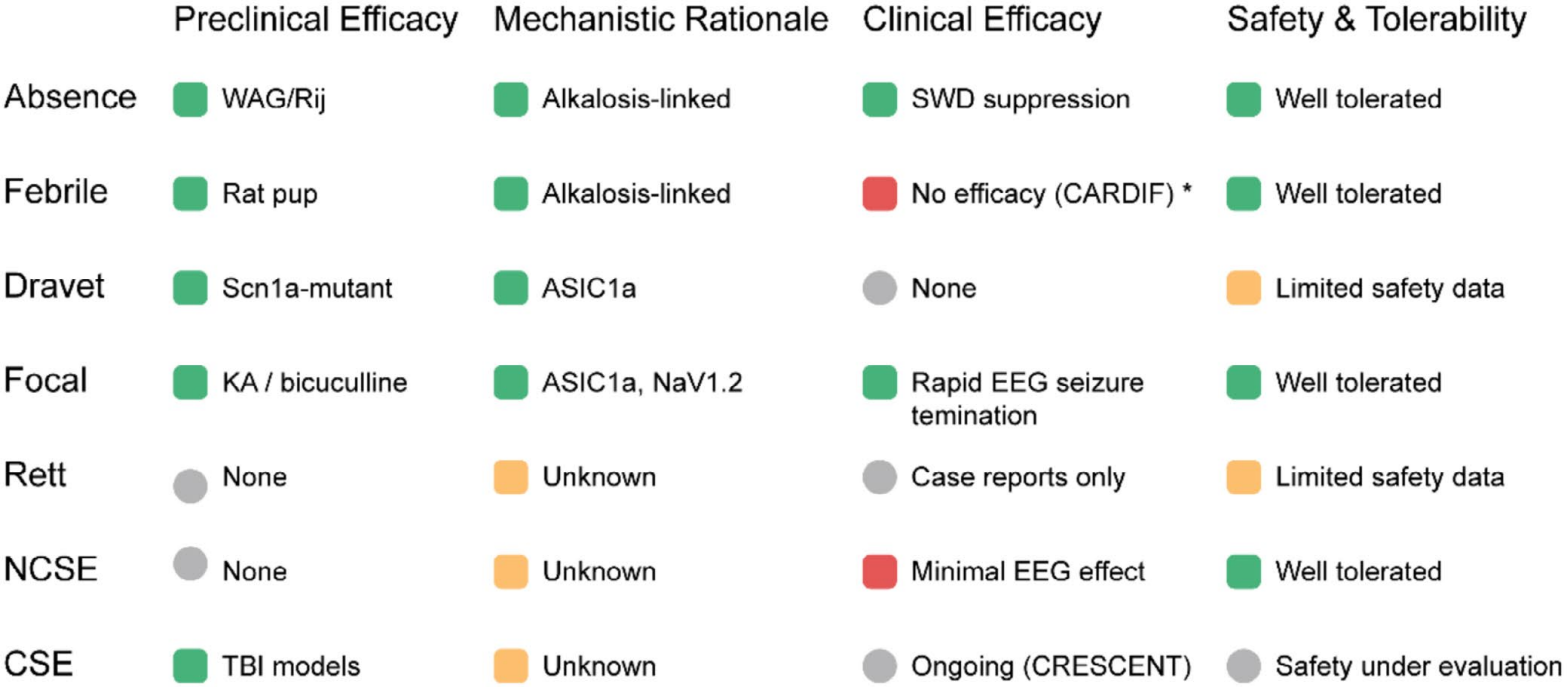
Translational profile of carbogen efficacy across major seizure types. Rows represent domains of evidence: preclinical data, mechanistic rationale, clinical outcomes, and safety profile. Color intensity reflects the strength or availability of evidence. Green: strong evidence; yellow: limited/inconsistent; red: no benefit or negative findings; gray: data not yet available. Preclinical and mechanistic data support robust effects in absence, focal, and Dravet syndrome models. However, clinical translation remains variable, especially in febrile seizures (CARDIF) and NCSE. *Notably, the CARDIF trial in febrile seizures reported no significant benefit over placebo; however, this outcome may reflect user‐side limitations (e.g., home‐based, parent‐administered setting) rather than mechanistic failure. The ongoing CRESCENT trial will further evaluate carbogen's role in acute pediatric CSE.

Overall, low‐concentration (5%) CO_2_ appears generally safe and mechanistically justified, yet its clinical efficacy varies markedly by seizure phenotype and user context. This inconsistency raises important questions about the interaction between biological mechanisms, disease states, and real‐world implementation, which are explored below. A comparative overview of the main clinical trials is provided in Table 4, with mechanistic insights summarized in Figure 2.

**TABLE 4:** Clinical trials evaluating 5% CO_2_ inhalation in seizure treatment.

References	Country	Study groups	Age	Seizure type	Protocol	Main outcomes
Tolner 2011 [10]	Finland	Self‐controlled, intraindividual comparison, *n* = 7	12–52	Drug‐resistant partial (focal) epilepsy	5% CO_2_ for 1 min post‐seizure	Rapid electrographic seizure termination
Yang 2014 [11]	China	Cross‐over design, self‐controlled, *n* = 12	4.8–13.4	Absence seizures	Hyperventilate with room air, 5% CO_2_, and 100% O_2_, each for 3 min	Reduction in SWDs; rapid seizure termination
Forsyth 2016 [14]	UK	Single‐arm trial, pre‐post EEG comparison, *n* = 6	3–13	NCSE	5% CO_2_ for 2 min post‐seizure	Transient EEG effects, no clinical improvement
Ramaraju 2021 [15]	UK	Secondary EEG re‐analysis, *n* = 5	3–13	NCSE	5% CO_2_ for 2 min post‐seizure	Highly variable EEG responses
Forsyth 2024 [17]	UK	Multicenter RCT (CRESCENT)	Pediatric	CSE	5% CO_2_ vs. placebo via face mask	Trial ongoing; registered (ISRCTN 52731862)
Weiß 2025 [16], Ohlraun 2013 [113]	Germany	Monocentric RCT (CARDIF); *n* = 93 (20 treated)	0.5–5	Febrile seizures	5% CO_2_ vs. placebo (100% O_2_) via parent‐operated face mask	Trial registered: NCT01370044; no significant difference in seizure termination between groups

*Note:* All trials were double‐blind and placebo‐controlled, and used short exposures (≤ 3 min).

Abbreviations: CSE, convulsive status epilepticus; NCSE, non‐convulsive status epilepticus; RCT, randomized controlled trial; SWDs, spike‐wave discharges.

### 3.3 Bridging Mechanistic and Clinical Gaps

CO_2_ responsiveness varies markedly across seizure types. Epilepsies with pH‐sensitive hyperexcitability, such as absence, febrile, or certain focal seizures, are generally more responsive to acidification [10, 11, 28]. In contrast, clinical benefit is limited in conditions whose pathophysiology frequently involves metabolic or structural disturbances rather than pH‐driven excitability. In an open‐label 5% CO_2_ trial for pediatric NCSE, only subgroups with receptor‐ or channel‐based dysfunction (e.g., Angelman, septo‐optic dysplasia) showed transient EEG improvement without clinical improvement, whereas metabolic (POLG1) and structural (PAFAH1B1) etiologies were largely unresponsive [14, 15]. This pattern suggests that network disorganization and impaired homeostatic buffering may blunt the inhibitory effects of CO_2_‐mediated acidification. The modest EEG modulation without clinical benefit may further indicate a dose–response threshold. In Scn1a (Nav1.1) Dravet models, 10% CO_2_ robustly suppresses seizures, whereas 5% CO_2_ does not [53], indicating that insufficient acidification, rather than mechanistic inadequacy, likely underlies these inconsistent clinical responses.

Translational discrepancies also arise at the implementation level. Although febrile seizures, a prototypical pH‐sensitive epilepsy, respond well to CO_2_ in controlled settings [28, 53], the CARDIF home‐use trial failed to reproduce these effects [16]. User‐dependent factors such as delayed seizure recognition, inaccurate device handling, premature termination of inhalation due to dyspnea or anxiety likely mitigated therapeutic benefit. These findings highlight that even in biologically responsive seizure types, clinical success depends more on dosing precision and patient tolerance than on mechanistic plausibility.

Taken together, inconsistencies between preclinical promise and clinical outcomes primarily arise from (1) etiologic and network heterogeneity determining pH responsiveness, and (2) operational and behavioral constraints limiting effective delivery. These insights define the boundary conditions for successful translation of CO_2_‐based therapy, while broader issues of safety and dose optimization are addressed in Section 4.

## 4 Current Translational Priorities and Future Directions

Despite strong mechanistic and preclinical evidence, carbogen therapy has yet to demonstrate consistent clinical efficacy, underscoring persistent barriers to translation. As discussed in Section 3.3, these discrepancies largely stem from etiologic and network heterogeneity shaping pH responsiveness [14, 15]. Such variability underscores the need for patient stratification and subtype‐specific targeting, since the same intervention may elicit distinct neurophysiological effects across genetic, metabolic, or structural epilepsies. Effectively bridging this heterogeneity represents the first step toward translating mechanistic plausibility into clinical relevance.

Beyond mechanistic diversity, practical and physiological limitations further constrain translation. Although low‐dose mixtures (≈5% CO_2_) are generally well tolerated under supervision [10, 11, 14], subjective respiratory sensations, dyspnea, air hunger and anxiety remain key barriers to adherence and dosing consistency [114, 115]. These perceptual effects, together with the gas's narrow therapeutic window, create a delicate balance between under‐ and over‐exposure: insufficient inhalation fails to achieve effective neural acidification, whereas stronger exposure risks hypercapnia or panic [116, 117]. Such constraints highlight the necessity of adaptive, closed‐loop regulation capable of dynamically titrating CO_2_ delivery to maintain efficacy while preserving comfort.

In addition, safety considerations extend beyond acute tolerability. While no serious adverse events have been reported in controlled contexts [81, 117, 118], the long‐term physiological and neurocognitive safety of repeated CO_2_ exposure remains insufficiently characterized. Potential risks include cumulative acidosis, cerebrovascular stress, and metabolic or cardiopulmonary complications [81, 82, 116]. Addressing these uncertainties will require systematic safety profiling and standardized monitoring, forming the empirical basis for individualized dosing protocols.

Together, these interrelated constraints, including mechanistic heterogeneity, limited tolerability, and unresolved safety profiles, constitute the principal translational barriers currently facing carbogen therapy. Addressing these limitations defines the next stage of investigation, centered on four immediate priorities for clinical advancement: (1) identifying and stratifying pH‐ and CO_2_‐responsive epilepsy subtypes; (2) developing adaptive, closed‐loop delivery systems integrating real‐time physiological feedback; (3) conducting dose‐ranging and longitudinal safety studies to determine evidence‐based exposure parameters; and (4) exploring rational combination with existing ASMs to optimize efficacy and synergy. Collectively, these priorities outline a coherent translational roadmap linking mechanistic insight, patient stratification, safety optimization, and technological innovation, topics developed in detail in Sections 4.1, 4.4.

### 4.1 Defining and Stratifying pH‐Sensitive Epilepsy Subtypes

Although pH sensitivity has emerged as a mechanistic feature across several epilepsy models, clinically actionable stratification criteria remain underdeveloped [119, 120]. Subtypes such as absence epilepsy, febrile seizures, Dravet syndrome, and TLE appear especially responsive to mild acidosis [10, 11, 46, 53], whereas NCSE often shows muted effects, potentially due to metabolic dysfunction and aberrant network maturation, as well as possibly involving impaired CO_2_ sensing or altered ion channel profiles [32, 121, 122].

Among molecular biomarkers, ASIC1a has drawn particular attention as a core mediator of pH‐dependent neuronal excitability that exhibits subtype‐specific expression differences across epilepsies. Its expression is upregulated in TLE but downregulated in focal cortical dysplasia [123, 124], suggesting that differential ASIC1a profiles may partly account for variable responsiveness to CO_2_‐based therapy. Importantly, the functional consequences of ASIC1a activation depend strongly on cell type and disease stage. Transient neuronal ASIC1a activation may facilitate inhibitory stabilization and seizure termination, whereas chronic astrocytic upregulation can enhance excitatory drive via glutamate release and extracellular acidification [12, 46, 47]. During chronic epilepsy, neuronal loss and reactive gliosis progressively redistribute ASIC1a expression from neurons to glia [125, 126], which may modify, but not necessarily reverse, the net physiological response to acidification [46]. Collectively, these findings indicate that ASIC1a expression level alone cannot reliably predict therapeutic responsiveness. Stratification frameworks should therefore integrate molecular, cellular, and temporal dimensions, recognizing that ASIC1a distribution, glial–neuronal balance, and disease stage jointly influence clinical outcomes.

Beyond ASIC1a, physiological parameters such as the hypercapnic ventilatory response (HCVR) and CO_2_‐evoked EEG changes offer non‐invasive metrics of central chemosensitivity and may predict treatment response [32, 127, 128]. Glial markers, such as glial fibrillary acidic protein (GFAP) or altered calcium dynamics, may further refine subtype classification, particularly in astrocyte‐driven seizure syndromes [55, 105, 129].

However, integrating these diverse biomarkers into clinical workflows remains challenging. Most preclinical data lack translation to human populations, and few prospective trials have linked molecular signatures to therapeutic outcomes [46, 130]. Table 5 summarizes current evidence and proposes a working model for stratifying pH‐sensitive epilepsy based on available markers and response patterns.

**TABLE 5:** Proposed pH responsiveness of epilepsy subtypes and supporting evidence.

Epilepsy subtype	Proposed pH sensitivity	Dominant circuitry	Representative etiology	Response to carbogen	Preclinical evidence	Clinical evidence
Absence epilepsy	High (hypocapnia‐triggered, alkalosis‐sensitive) [18]	Thalamo‐cortical loops	Idiopathic (e.g., Childhood Absence Epilepsy)	Strong (✓✓✓)	WAG/Rij: SWD suppression [18]	Pilot pediatric EEG: SWD aborted by 5% CO_2_ [11]
Febrile seizures	High (alkalosis‐triggered) [131, 132]	Cortico–hypothalamic interaction	Fever‐induced (e.g., typical febrile)	Strong (✓✓✓)	Rat pups: CO_2_ rapidly terminates seizures [28]	Pilot pediatric trial: no significant benefit over placebo [16]
Dravet syndrome	Moderate (CO_2_ chemosensitivity deficit) [21, 32]	Brainstem + forebrain (RTN, amygdala)	SCN1A mutation	Moderate (✓✓)	*Scn1a* ^*+/−*^ mice: CO_2_ suppresses seizures [46, 53]	No formal clinical trial
Focal epilepsy	Moderate (local alkalosis‐sensitive) [12]	Cortical focus (e.g., hippocampus, neocortex)	Structural lesion, sclerosis	Moderate (✓✓)	Various rodent/macaque models: CO_2_ reduces spikes and seizure freq. [10, 29]	Pilot adult EEG: rapid suppression in drug‐resistant focal epilepsy [10]
Rett syndrome	Moderate (impaired CO_2_ response) [133, 134]	Brainstem + autonomic nuclei	MECP2 mutation	Moderate (✓✓)	Limited preclinical data	Case reports: Carbogen + pipamperone reduced apneas (via alkalosis correction and 5‐HT modulation) [135]
NCSE	Low/mixed (unclear pH modulation)	Diffuse cortical/subcortical networks	Mixed etiologies	Minimal (✓)	No specific models	Open‐label: safe but minimal effect [14, 15]
CSE	Unknown (possibly low in prolonged seizures) [136]	Brain‐wide, frontotemporal + subcortical	Various (e.g., trauma, infection, toxin)	Unclear (✓)	Limited preclinical data	Phase II pediatric trial ongoing [17]

*Note:* This table summarizes hypothetical pH sensitivity, circuit‐level pathophysiology, and available evidence for the effect of CO_2_ or carbogen in selected epilepsy subtypes. −, no evidence; ✓, weak evidence; ✓✓, moderate evidence; ✓✓✓, strong evidence.

Abbreviations: CSE, convulsive status epilepticus; NCSE, non‐convulsive status epilepticus.

Looking ahead, a multidimensional stratification framework combining functional imaging, blood‐based biosensors (e.g., exosomal ASIC1a mRNA), wearable capnography, and machine learning analytics may enable real‐time identification of responsive patients [137, 138, 139, 140, 141]. Such tools will be essential for selecting appropriate candidates for carbogen and related interventions.

### 4.2 Toward Personalized pH‐Modulating Interventions

Substantial interindividual variability in carbogen response reflects differences in genetic background, neuronal pH sensitivity, respiratory dynamics, and comorbid conditions. These differences necessitate a move toward personalized, physiology‐informed dosing protocols. Emerging closed‐loop systems, integrating real‐time EEG, capnography, and physiological monitoring, offer a promising platform for individualized carbogen delivery. By coupling AI‐based seizure prediction with feedback from wearable sensors, these systems may adjust dosing dynamically to optimize efficacy and safety [137, 142, 143].

Physiological stratifiers, such as HCVR and end‐tidal CO_2_ (EtCO_2_) dynamics, can help identify patients with impaired CO_2_ clearance or altered chemosensitivity, guiding both eligibility and dosing [33, 144]. New biotechnologies are also advancing autonomous pH‐responsive platforms. For example, optogenetic constructs gated by intracellular acidification have been shown to suppress seizures in preclinical models, offering a proof‐of‐concept for cell‐intrinsic control of excitability [145]. Likewise, biocompatible, wearable pH and CO_2_ biosensors, potentially linked to telehealth infrastructure, may facilitate safe, at‐home monitoring and delivery, particularly in pediatric or resource‐limited settings [146, 147].

Despite these advances, clinical translation remains limited by insufficient validation in real‐world settings, especially in patients with comorbid respiratory or psychiatric vulnerabilities [116, 148, 149]. Current systems often lack the reliability and usability required for unsupervised use. A phased clinical development model is needed, beginning with feasibility studies in high‐control environments, followed by stratified multicenter trials that incorporate physiological and biomarker data into dosing algorithms. Patient and caregiver feedback should inform device refinement and delivery workflows, ensuring both safety and user acceptability in at‐home settings [150, 151]. Ultimately, integrating personalized monitoring with adaptive delivery systems represents a key frontier in carbogen therapy, one that bridges mechanistic neuroscience with practical neurology.

### 4.3 Clinical Safety, Tolerability, and Dose Optimization

Preliminary studies suggest that 5% CO_2_ is generally well tolerated in patients with epilepsy under supervision, with no serious adverse events reported [10, 123]. However, systematic large‐scale evaluations of safety profiles, patient‐reported tolerability, and dose–response parameters are still lacking, particularly across different epilepsy subtypes and vulnerable populations. This gap hampers the establishment of evidence‐based exposure limits and standardized clinical protocols. Unlike systemically administered ASMs, carbogen's inhalational delivery can evoke acute perceptual sensations, such as dyspnea, air hunger and anxiety that influence adherence and subjective tolerability [81, 117, 118]. Recognizing these challenges is critical to realistically define carbogen's clinical role rather than downplaying its limitations. Owing to its overt respiratory effects and narrow therapeutic window, carbogen is currently deemed unsuitable for unsupervised or at‐home use [16]. Additionally, at higher concentrations or with prolonged exposure, there remains a potential risk of CO_2_ narcosis [27], further emphasizing the necessity for strictly supervised administration and precise dose titration in monitored clinical settings.

Future development should focus on systematically improving the safety–tolerability interface. Structured administration protocols integrating real‐time physiological monitoring, such as capnography, end‐tidal CO_2_, and pH tracking, could help maintain efficacy while minimizing perceptual distress. Wearable sensors and closed‐loop systems that dynamically adjust delivery parameters based on physiological feedback represent promising tools for individualized control [142, 152]. Desensitization‐based acclimation and simplified interface designs may further enhance comfort and compliance, particularly in pediatric or cognitively impaired groups.

Beyond acute tolerability, long‐term physiological and neurocognitive safety remains insufficiently characterized. Repeated or prolonged CO_2_ exposure may lead to cumulative acidosis, CO_2_ retention, or cerebrovascular alterations, especially in individuals with reduced ventilatory reserve, metabolic disorders, or cardiovascular comorbidities [81, 82, 116]. Comprehensive baseline profiling, including assessment of bicarbonate, creatinine, calcium, and urea nitrogen, can help identify metabolic susceptibility [148, 153, 154], while biomarkers such as IL‐1β, GFAP, and heart rate variability (HRV) may signal neuroinflammatory or cardiovascular stress [55, 105]. Longitudinal neurocognitive assessment is also warranted to evaluate potential effects on learning, attention, and executive function in developing or aging populations [155, 156, 157].

Establishing a reliable safety framework is therefore both an immediate and future priority. Standardized monitoring protocols, longitudinal neurocognitive follow‐up, and multicenter safety registries are needed to define safe exposure limits, refine patient eligibility, and optimize dosing algorithms [153, 158]. Together, these measures will facilitate the transition of carbogen from a promising experimental therapy to a clinically viable, precision‐tailored intervention.

### 4.4 Integration With Conventional ASMs

Rational integration of carbogen with conventional ASMs represents a promising yet underexplored direction in epilepsy therapy. Carbogen provides rapid, physiology‐based seizure termination, whereas ASMs sustain long‐term stabilization through systemic modulation. Combining both may achieve complementary temporal control, immediate suppression by CO_2_, followed by prolonged maintenance, potentially reducing cumulative ASM exposure and limiting tolerance or dose‐related adverse effects. Although direct clinical data on carbogen–ASM combinations remain limited, preclinical evidence indicates that carbonic anhydrase inhibitors (CAIs) can synergize with lamotrigine (LTG) or levetiracetam (LEV) to enhance seizure suppression while maintaining tolerability [159, 160, 161]. This mechanistic complementarity suggests that carbogen, acting through brief and reversible acidification, might achieve comparable synergy without compromising the efficacy or safety of ASMs.

Pharmacologically, the principal concern involves bidirectional physiological interaction between carbogen and concurrent ASM therapy. Carbogen‐induced hypercapnia transiently perturbs acid–base balance and ventilatory regulation, thus necessitating careful coordination with agents that affect respiration or metabolism. For instance, benzodiazepines (BZDs), the first‐line agents for acute seizure termination in SE, can suppress respiratory drive [162]; consequently, co‐administration with carbogen requires close monitoring of oxygen saturation, end‐tidal CO_2_, and respiratory rate to prevent CO_2_ retention and respiratory compromise. This strategy is prospectively evaluated in the ongoing CRESCENT trial, which is assessing carbogen as an adjunct to BZD therapy for pediatric CSE [17]. Similarly, CAIs such as topiramate (TPM) or acetazolamide (AZM), which are especially effective in absence epilepsy, catamenial epilepsy, and certain metabolic or channelopathy syndromes, contribute to seizure control by inducing mild metabolic acidification [157, 163, 164]; concurrent CO_2_ inhalation may potentiate this shared acidifying mechanism transiently, necessitating individualized dosing and physiological supervision [165]. In contrast, both LTG, which does not significantly alter CO_2_ chemosensitivity [166], and LEV, which stabilizes mitochondrial redox status without perturbing systemic pH [167, 168], demonstrate particular compatibility with carbogen‐based adjunctive therapies. Table 6 summarizes overlapping molecular targets and pH‐sensitive mechanisms, providing the mechanistic rationale for combined approaches.

**TABLE 6:** Comparison of mechanistic targets between carbogen and representative ASMs.

Mechanism of action	Carbogen	ASMs (e.g., VPA, LEV, TPM)	Interaction type
Brain pH modulation	✔ (via respiratory acidosis) [13]	✖ (except TPM: mild CA inhibition) [157]	Complementary
ASIC1a activation	✔ (acid‐induced activation) [12]	✖ (no direct effect reported)	Unique
Na^+^ channel subtype modulation	✔ (NaV1.2 inhibition under acidosis) [13]	✔ (e.g., LTG, CBZ: broad VGSC blockade) [169]	Synergistic
K^+^ channel activation	✔ (KATP‐mediated hyperpolarization) [55]	∼ (e.g., ezogabine, withdrawn) [169]	Complementary
Glutamate receptor inhibition	✔ (via acidosis‐mediated NMDA antagonism) [59]	✔ (e.g., felbamate, TPM) [169]	Synergistic
GABAergic transmission enhancement	✔ (via pH potentiation) [62]	✔ (e.g., VPA, BZDs) [169]	Synergistic
Adenosine signaling enhancement	✔ (via reduced uptake, ↑ extracellular adenosine) [69]	✖ (no direct effect reported)	Unique
Neuroinflammation and oxidative stress suppression	✔ (↓ gliosis, ROS, cytokines in rodents) [97]	∼ (e.g., mild with VPA) [170]	Synergistic
Cerebral perfusion enhancement	✔ (↑ CBF via vasodilation) [17]	✖ (no direct effect reported)	Complementary
Respiratory effects	↑ ventilation, oxygenation [11]	↓ respiratory drive (e.g., BZDs) [17]	Complementary

*Note:* ∼, unclear or inconsistent evidence; ✔, present/confirmed; ✖, absent/not reported. Interaction types: complementary, additive effects via distinct pathways; synergistic, potentially convergent mechanisms, evidence suggests enhancement; unique, mechanism only targeted by carbogen.

Abbreviations: ASMs, antiseizure medicines; BZDs, benzodiazepines; CBF, cerebral blood flow; CBZ, carbamazepine; LEV, levetiracetam; LTG, lamotrigine; TPM, topiramate; VGSC, voltage‐gated sodium channels; VPA, valproic acid.

Nonetheless, empirical validation remains imperative prior to clinical translation. Building on preliminary evidence of CO_2_‐mediated pH modulation and ASM pharmacodynamics [159, 161], future research should prioritize pharmacodynamic and metabolic profiling to delineate ASM–CO_2_ interactions, including dose sequencing, acid–base equilibrium regulation, and respiratory feedback mechanisms. Preclinical combination models could clarify whether effects on excitability and metabolism are additive versus antagonistic. Concurrently, controlled clinical studies with continuous pH and ventilatory monitoring will be essential to establish safety parameters and minimize the risk of reduced efficacy or enhanced toxicity when carbogen is used as an adjunct. Such evidence will define the role of carbogen as an acute adjunct within multimodal epilepsy therapy.

**FIGURE 3:**
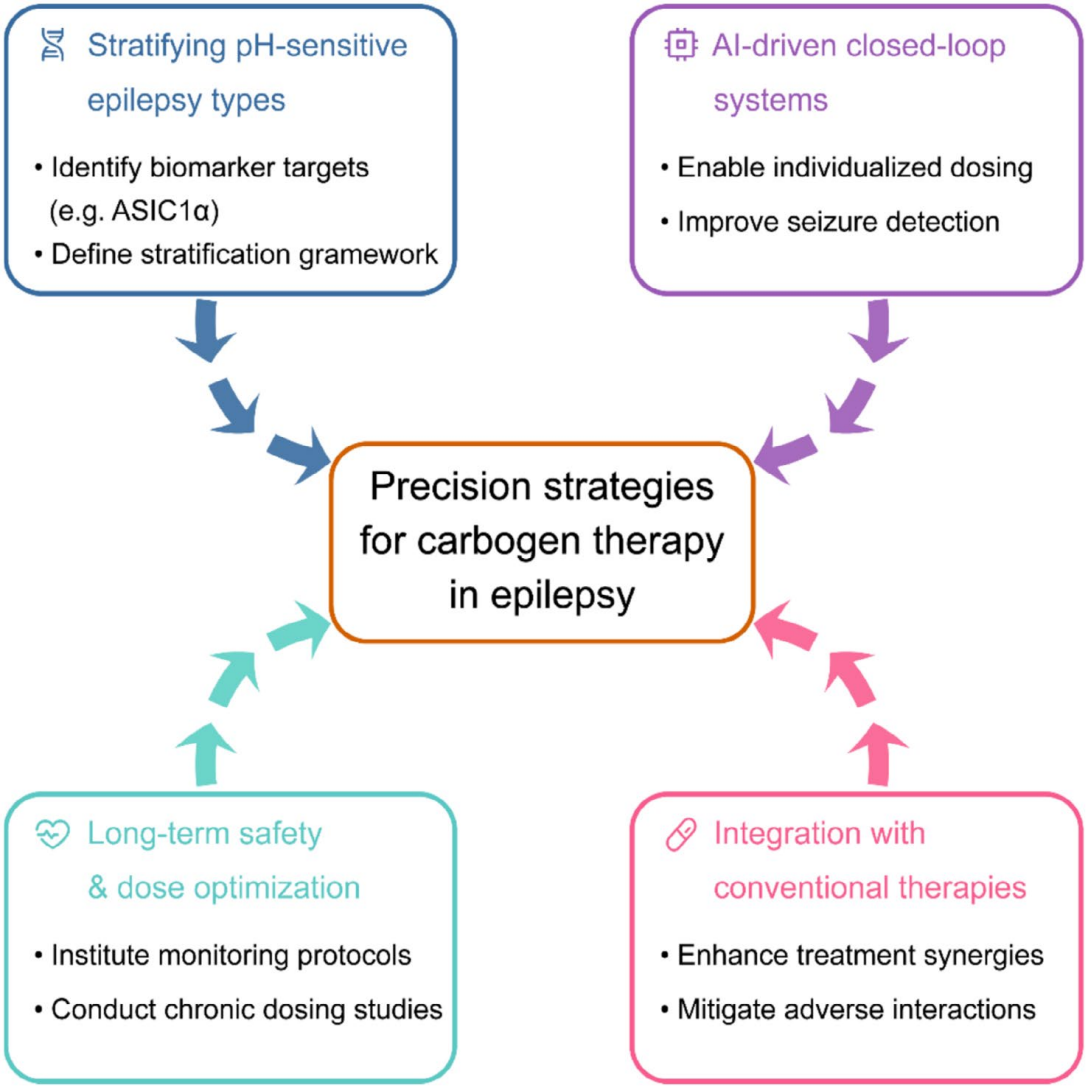
A multidimensional framework for the precision application of carbogen therapy in epilepsy. This schematic summarizes four core strategic directions for advancing carbogen therapy: (1) biomarker‐guided identification of pH‐sensitive epilepsy phenotypes, (2) individualized dosing via AI‐based closed‐loop delivery systems, (3) long‐term safety optimization through metabolic and inflammatory monitoring, and (4) rational integration with conventional ASMs. These strategies aim to enhance efficacy, reduce toxicity, and support widespread adoption of carbogen as a personalized rescue intervention.

Together, these future directions provide a multidimensional framework for the ongoing refinement of carbogen therapy, including patient stratification, delivery optimization, safety evaluation, and clinical integration. As summarized in Figure 3, an integrated approach that links mechanistic insights with precision tools could support the translation of carbogen from an experimental intervention toward a targeted adjunct in selected epilepsy populations.

## 5 Conclusion

Carbogen inhalation represents a promising, non‐pharmacological strategy for rapid seizure termination, particularly in focal and absence epilepsy. Its efficacy arises from acute modulation of pH‐sensitive neuronal and glial excitability, distinguishing it from conventional ASMs. However, heterogeneous treatment responses across epilepsy subtypes, and the challenges of consistent administration outside controlled clinical settings, highlight the need for individualized, precision‐based approaches. To optimize therapeutic outcomes, future strategies should incorporate electroclinical phenotyping, respiratory‐metabolic profiling, and neuroinflammatory biomarkers to guide patient selection. Advances in rapid biomarker detection and AI‐assisted risk modeling may enable the development of closed‐loop carbogen delivery systems that dynamically adjust dosing based on real‐time physiological feedback. Future research should focus on stratified clinical trials, integration with standard ASMs, and systematic long‐term safety assessments. Transitioning carbogen from experimental rescue therapy to a validated clinical intervention will require coordinated progress in translational research, real‐world implementation, and incorporation into personalized neurology frameworks tailored to pH‐sensitive epilepsy syndromes.

## Funding

This work was supported by the Hubei Provincial Department of Education Scientific Research Plan for Young Talent Project (Q20234306) and the Scientific Research Project of Jingchu University of Technology (YY202415).

## Conflicts of Interest

The authors declare no conflicts of interest.

## Data Availability

Data sharing is not applicable to this article as no new data were created in this study. The authors confirm that the data supporting the findings of this study is available within the article.
